# Sexual size dimorphism in anurans fails to obey Rensch’s rule

**DOI:** 10.1186/1742-9994-10-10

**Published:** 2013-03-09

**Authors:** Wen Bo Liao, Yu Zeng, Cai Quan Zhou, Robert Jehle

**Affiliations:** 1 Key Laboratory of Southwest China Wildlife Resources Conservation (Ministry of Education), China West Normal University, Nanchong, Sichuan 637009, China; 2 Institute of Rare Animals and Plants, China West Normal University, Nanchong, Sichuan 637009, China; 3 School of Environment & Life Sciences, University of Salford, Salford M5 4WT, UK

**Keywords:** Allometry, Anurans, Rensch’s rule, Sexual size dimorphism

## Abstract

**Background:**

Sexual size dimorphism (SSD) is related to ecology, behaviour and life history of organisms. Rensch’s rule states that SSD increases with overall body size in species where males are the larger sex, while decreasing with body size when females are larger. To test this rule, we analysed literature as well as own data on male and female body size in anurans (39 species and 17 genera). We also tested the hypothesis that SSD is largely a function of age difference between the sexes.

**Results:**

Our data set encompassed 36 species with female-biased SSD, and three species with male-biased SSD. All considered species failed to support Rensch’s rule, also when the analyses were phylogenetically corrected. However, SSD was significantly correlated with Sexual Age Difference (SAD) across species. We also found a significant correlation between SSD contrasts and SAD contrasts.

**Conclusions:**

Our study suggests that Rensch’s rule does not accurately describe macroevolutionary patterns of SSD in anurans. That SAD can explain most of the variation in SSD among species when controlling for phylogenetic effects suggests that phylogeny is not responsible for the broad relationship between age and size across the sexes.

## Introduction

Sexual size dimorphism (SSD) is related to the ecology, behaviour and life history of organisms, and widely observed in the animal kingdom [1,2]. In most invertebrates and ectothermic vertebrates, females are larger than males, whereas in endothermic animals males are generally larger than females [1,2]. Beyond these broad generalisations, the degree of SSD is however highly variable within and between taxa. Rensch [3] proposed that SSD increases with overall body size in species where males are the larger sex, and decreases with body size in species where females are the larger sex. This pattern of variation, known as Rensch’s rule, has been confirmed in a wide range of taxa including flower mites [4], water striders [5], dragonflies [6,7], lizards [2,8,9], snakes [2], turtles [10], hummingbirds [4,11], primates [12,13], shorebirds [14], drosophilid flies [15], salmonid fishes [16], grouse [11], cattle breeds [17], domestic goats and sheep [18] and bustards [11,19], although a range of examples for opposing patterns also exist both at the inter- and intraspecific level [20-25].

Several hypotheses have been proposed to explain the evolution of SSD. Firstly, theory predicts that the intensity of sexual selection in a given sex is a driver of body size evolution [20,25]. Sexual selection is generally thought to favour larger males, and should therefore result in male-biased SSD [26]. Female-biased SSD, on the other hand, can arise in species where females strongly compete for mates [14]. Secondly, fecundity selection towards higher allocation of resources to individual offspring, the production of higher numbers of offspring, or the ability to reproduce more frequently can also increase the size of females, and has been hypothesized to be related to the inverse of Rensch’s rule [8,27]. Thirdly, correlational selection predicts that strong selection on size in one sex also affects the opposing sex, revealing a phenotypic coupling for size between the sexes among related species, as well as among populations within species [2]. When sexual and fecundity selection are unequal or one of them is lacking, then the stronger force will drive size divergence between populations or species through the respective sex, while the other sex will change at a slower pace due to genetic correlations between sexes. Finally, the life-history hypothesis predicts that differences in age and growth between males and females may further contribute to SSD, explaining allometry of SSD following Rensch’s rule as the proximate mechanism [15,16].

In most (but not all) cases, allometry in male-biased SSD follows Rensch’s rule at the interspecific and intraspecific level [2,13,23,28]. However, the trend is questionable in species with female-biased SSD [22-24]. In anurans, sexual size dimorphism is generally female-biased [29]. Although this can be explained by natural selection for optimal body size [30], directional selection for larger males [26], and differences in age structure between sexes [31,32], no information about the consistency of allometry for SSD with Rensch’s rule is currently available. In this study, we provide one of the first interspecific tests of Rensch’s rule in anurans, using data from 39 species markedly differing in body size. Our aims were to: (1) examine patterns of SSD across 39 anurans species; (2) test the consistency of allometric relationships with Rensch’s rule; and (3) test the hypothesis that variation in SSD is a function of sexual age differences (SAD).

## Results

**Figure 1:**
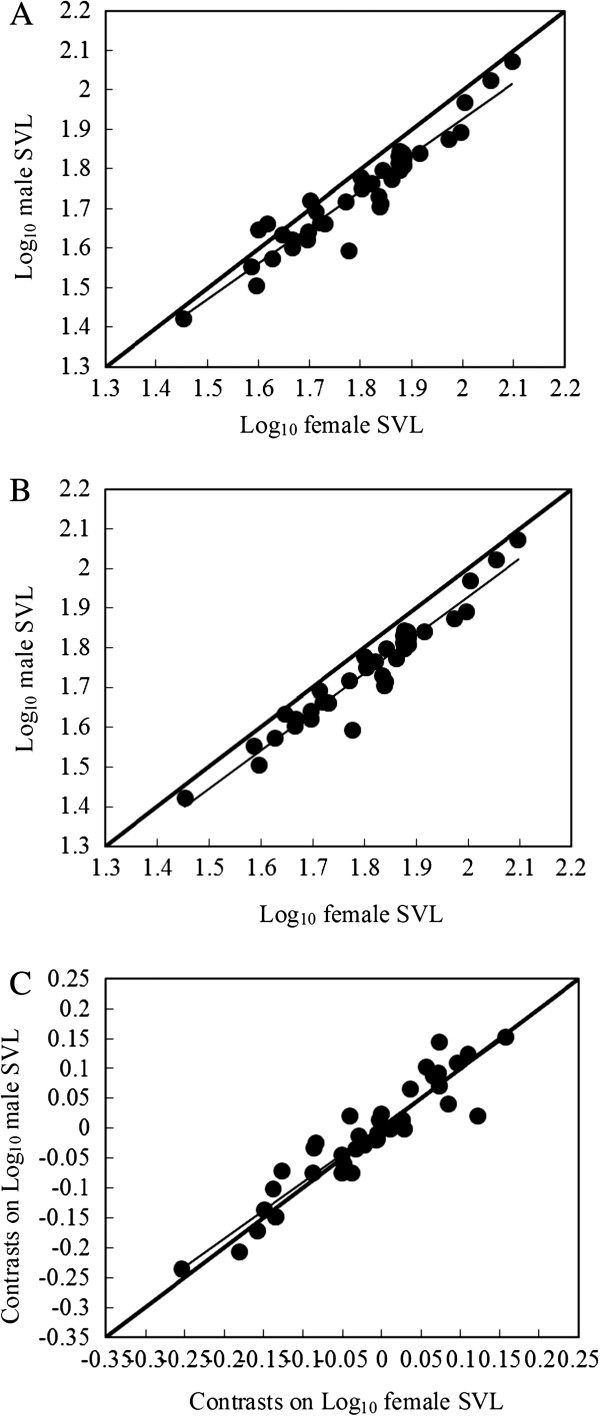
**Allometry of SSD in anurans.** The linear (Model I) regression line (β) with 95% confidence interval (weak line) is shown for SSD. The thick grey line represents isometry, i.e. β = 1. Each dot represents one species based on the mean body size of males and females. **A**: SSD-size relationship for 39 species, Model I, β = 0.992; **B**: SSD-size relationship for 36 species with female-biased SSD, Model I, β = 0.967; **C**: SSD-size relationship for 36 species with female-biased SSD calculated from the phylogenetic tree in Figure 3, using independent comparisons (Felsenstein [62]; Garland et al. [66]), Model I, β = 0.974).

The degree of SSD differed significantly among species and sexes (species, *F*_38, 77_ = 64.992, *P* < 0.001; sex, *F*_1, 77_ = 38.411, *P* < 0.001; species × sex, *F*_38, 77_ = 9.421, *P* < 0.001). Thirty-six species were characterised by female-biased SSD, whereas male-biased SSD was found in 3 species. Variation in SSD did not follow Rensch’s rule (Figure 1A). Model I revealed a significant isometric relationship between the mean size of the sexes across 39 species (i.e. β = 1 was not rejected; *F*_1, 38_ = 343.374, *R*^2^ = 0.903, β = 0.992 ± 0.054, 95% CI = 0.938-1.046, *P* < 0.001). Model II revealed a similar slope (β = 1.044 ± 0.057, 95% CI = 0.987-1.101, *P* < 0.001). Phylogenetically corrected analyses also showed that variation in SSD did not support the existence of Rensch’s rule (β = 0.974 ± 0.082, 95% CI = 0.892-1.056, *P* < 0.001; Figure 1B). RMA regression for the analysis of contrasts showed a similar relationship (β = 0.958 ± 0.076, 95% CI = 0.882-1.034, *P* < 0.001).

Species with female-biased SSD did not follow Rensch’s rule, as SSD did not decrease with body size (Figure 1C). Model I revealed a significant relationship between male and female size (*F*_1, 35_ = 484.223, *R*^2^ = 0.934, β = 0.967 ± 0.044, 95% CI = 0.923-1.011, *P* < 0.001), similar to what is assumed under isometry (test of hypothesis: β = 1). Model II revealed a similar slope (β = 0.996 ± 0.043, 95% CI = 0.953-1.039, *P* < 0.001). Variation in SSD did not support the existence of Rensch’s rule when controlling for phylogenetic effects (Model I: β = 0.935 ± 0.051, 95% CI = 0.870-1.000, *P* < 0.001; Model II: β = 0.948 ± 0.043, 95% CI = 0.892-1.002, *P* < 0.001), indicating that the lack of significance might be due to low sample size.

**Figure 2:**
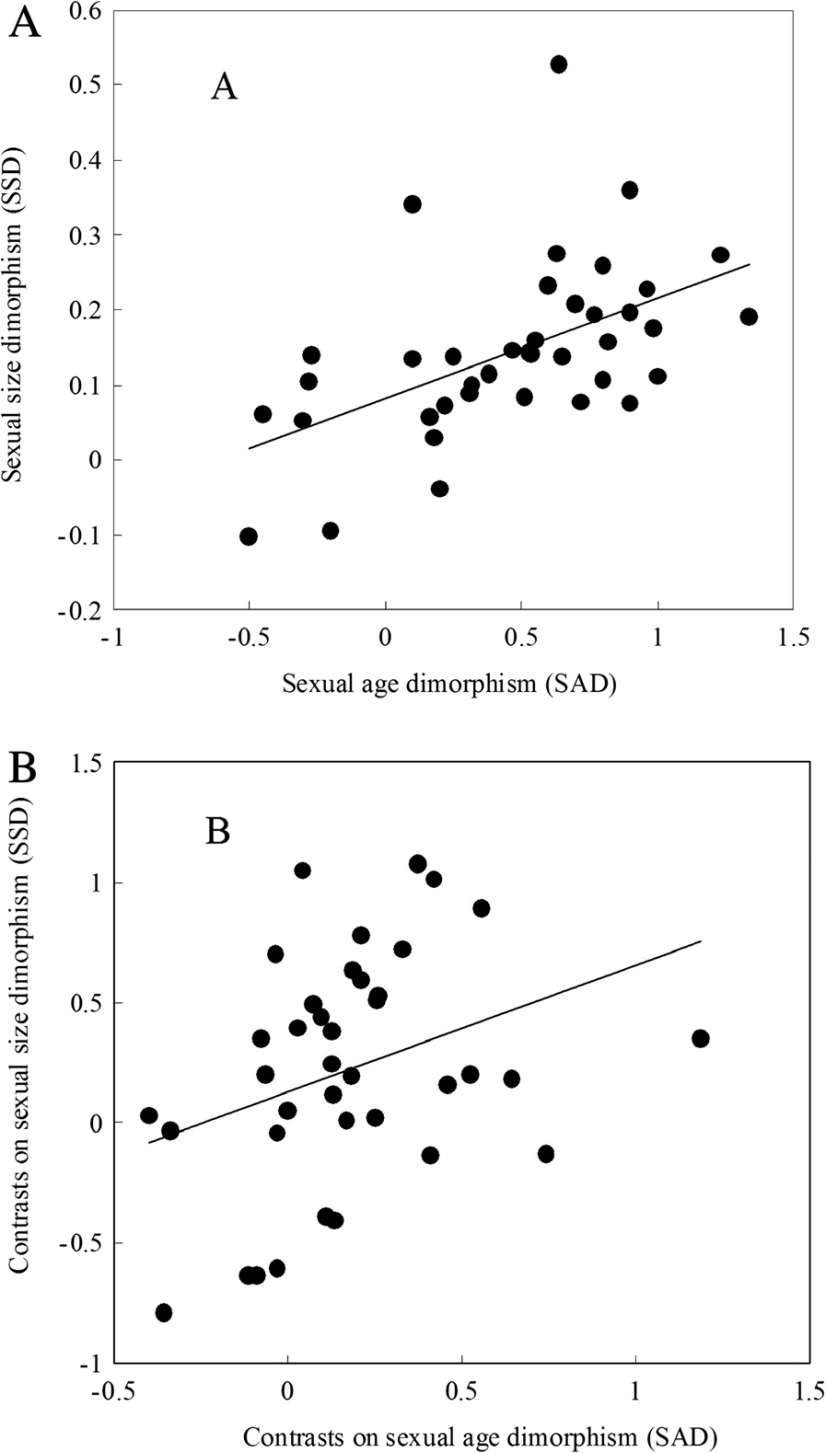
**The relationship between SSD (Log (female mean size) –Log (male mean size) ratio) and SAD (Log (female mean age)–Log (male mean age) difference) for 39 anurans species. Each dot represents a species. A**: a significant correlation between SSD and SAD (*r* = 0.520, *P* = 0.001); **B**: a significant correlation between SSD contrasts and SAD contrasts calculated from the phylogenetic tree in Figure 3, using the method of independent comparisons (Felsenstein [62]; Garland *et al.*[66]) (*r* = 0.339, *P* = 0.037).

**Figure 3:**
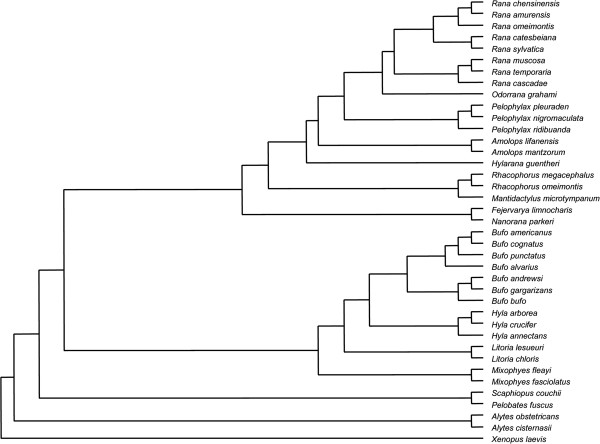
**The phylogenetic tree of the anuran used in the comparative analysis following Frost et al.**[ [65]]**.**

No deviation from normal distribution was found for both SSD and SAD (one-sample Kolgomorov–Smirnov two-tailed test: SSD, Z = 0.781, *P* = 0.657; SAD, Z = 0.721, *P* = 0.677). SSD was significantly correlated with SAD across species (Pearson correlation analysis: *r* = 0.520, *P* < 0.001, *n* = 39; Figure 2A). The set of SSD and SAD contrasts obtained from the phylogenetic tree (Figure 3) also did not deviate significantly from a normal distribution (one-sample Kolgomorov–Smirnov two-tailed test on SSD and SAD contrasts: Z = 0.499, *P* = 0.965 and Z = 0.834, *P* = 0.484, respectively). Residuals showed no heteroscedasticity (Pearson correlation coefficient between residuals and predicted SSD values: *r* = −0.038, *P* = 0.830) and were normally distributed (one sample Kolgomorov–Smirnov two-tailed test: Z = 0.588, *P* = 0.879). A correlation analysis between SSD and SAD contrasts through the origin (Figure 2B) was significant (*r* = 0.339, *P* = 0.037, *n* = 38), confirming that SSD contrasts can be largely explained by SAD contrasts.

## Discussion

Rensch’s rule has been widely confirmed in various animal taxa [2,8,9,17,18,26,27,33]. The majority of studies found that sexual selection on male size is satisfactory at explaining Rensch’s rule [8,9,14,26-28,34]. However, Rensch’s rule for example persists in domestic mammals, for which sexual selection has become replaced by human control [17,18]. In a few cases, fecundity selection has been effective at explaining the inverse of Rensch’s rule [2,24,35]. Our study on anurans showed that SSD-size relationships in anurans were inconsistent with Rensch’s rule and the inverse of it, because species with female-biased SSD displayed isometric relationships with mean body size.

Rensch’s rule is nearly universal among taxa with male-biased SSD. In taxa with female-biased SSD, either Rensch’s rule or the inverse of it are regularly demonstrated [2,23,36]. Patterns failing to support Rensch’s rule or its inverse have been found exclusively in taxa with female-biased SSD [2,21,22]. The results of our inter-specific study conform to this pattern. To explain the lack of association between SSD and size, we suggest that fecundity selection on females favouring large size (which is supposed to be strong in ectotherms) balances out sexual selection in favour of large male size.

Variation in SSD is affected by factors other than sexual, correlational and fecundity selection, including life-history traits as well as energetic and ecological constraints. Previous studies showed that SSD in anurans can be explained by age differences between males and females [16,32,37-49]. Our results demonstrate a correlation between SSD and SAD across 39 species, identifying SAD as a main factor contributing to the extent of SSD across species. Similarly, developmental time differences between the sexes previously explained the extent of SSD in arthropods [2,15] and fish [16].

SSD results from sexual divergence in ontogenetic trajectories [50-52], which may reflect a trade-off in energy allocation between somatic growth, survival and reproduction [53]. Based on the functional relationship of adult body size = *f* (initial size + growth rate × age) [44], the adult size of a given sex depends on growth duration in combination with growth rate. Like most ectothermic animals with indeterminate growth, the age at maturity is a critical life-history trait to determine the ontogenetic trajectory in anurans. Males usually mature earlier than females [54], resulting in a female-biased SSD because a later maturation leads to more energy being devoted to somatic growth to achieve large body size [44,54]. Growth rates also contribute to body size and thus to SSD. SSD in anurans is positively correlated with sex difference in age at maturity, and negatively correlated with the corresponding difference in annual growth rate, with the relative contributions of age and growth to SSD varying among species [52]. This ontogenetic pattern supports our result that SSD did not obey Rensch’s rule. Energy is the ultimate basis underlying the growth divergence between sexes when selection favours males and females to reach differential optimal sizes. For anurans with female-biased SSD, strong fecundity selection enhances female investment in offspring production, constraining their potential for growth [52]. Males could be less affected from such constraints because, compared to females, reproduction is significantly less costly for them [52,53].

Ecological factors usually operate through ontogeny in sex-specific ways to affect the body size of individuals and species [55-57]. Diet and temperature can induce substantial phenotypic plasticity in body size of ectothermic animals. In general, animals reared on lower quality diets mature smaller [58,59], and animals reared at lower developmental temperatures mature larger [60]. Variation in SSD can arise when males and females respond differently to diet or temperature (differential- plasticity hypothesis) [61]. For anuran species with an allometric relationship between overall size and trophic morphology and/or differential temperature preferences between sexes, SSD can be associated with intersexual niche partitioning [30,32].

Treating species as independent units yielded in results which are qualitatively different from those using phylogenetically independent contrasts. SSD contrasts can be explained by SAD contrasts because the relationships between female and male size and age may not be influenced by phylogenetic relatedness. Similarly, variation in SSD in 17 anurans has previously been explained through SAD based on phylogenetic information incorporating independent contrasts [32].

Like in previous studies [25,35], methodological aspects give reason to view our results with caution. Mean age and size may vary considerably between years in the same population. Skeletochronology might also be problematic, because endosteal resorption and false lines can affect age estimation. However, any bias in estimating individual age should equally affect males and females. Results of correlations across species should also be regarded cautiously because species’ data points cannot be assumed to be statistically independent [61,62]. However, Harvey and Pagel [63] pointed out that comparisons across species still lead to meaningful analyses unless they depend on a cluster of points that share an immediate common ancestor.

## Methods

We obtained sex-specific demographic and morphological data on mean age and size across 39 species and 17 genera from the literature and our own data based on species from the Sichuan Province, China (Additional file 1: Table S1). Individuals were collected by hand at night using a flashlight. Sex was determined based on secondary sexual characteristics (vocal sacs in adult males, and eggs readily visible through the abdominal skin in adult females). We measured body size (snout-vent length, SVL) of each individual using a calliper, holding each individual in its normal posture. We stored the second phalange of the hind limb in 10% neutrally buffered formalin for skeletochronology. Some individuals were subsequently released at the point of capture, whereas other individuals were brought to the lab for studying testis size and sperm traits. Individual age was estimated by skeletochronology as follows. We removed the skin and muscle tissues of each digit, and decalcified the remaining bones in 5% nitric acid for 48 h before washing them in running tap water for 24 h. We then stained the decalcified digits for 150 min in Haris’s haematoxylin and rinsed them with distilled water. Subsequently, we dehydrated the stained bones through successive stages of increased ethanol concentrations (1 h in each concentration). We cross-sectioned the diaphyseal region of each phalanx at a thickness of 8 μm and selected the smallest medullar cavity of the sections to examine LAGs (Lines of Arrested Growth) with a LEITZ dialux 40 microscope, photographing the sections using a Motic BA300 digital camera mounted on a Moticam2006 light microscope at × 400 magnifications. Endosteal resorption of long bones starts from the inner surface of the bone, enlarging the marrow cavities and eroding a portion of LAGs after hibernation [40]. We confirmed the first LAG based on the Kastschenko Line (KL; the interface between the endosteal and periosteal zones) [41,42]. False and double lines were rarely observed and not considered as true LAGs in all samples. Following Monnet and Cherry [32], we calculated the mean values for the population as algebraic means for each year, weighted by sample size. Mean values for species were obtained as algebraic means of population values regardless of the sample size in cases where data were available for different populations. We calculated SAD as the difference between log-transformed female and male mean ages because age is strongly heteroscedastic. Following Lovich and Gibbons [64], SSD was calculated as (log (female mean size)/ log (male mean age)) - 1, arbitrarily set positive when females are larger and negative when males are larger.

For our comparative analysis, we used an established phylogeny [65] (Figure 3). We calculated mean size and age in both sexes for ancestral nodes as the algebraic mean of the two closest lower nodes [61]. Details of the general procedure for estimating the character values in the ancestors are presented in Felsenstein [62]. With 39 species at the tips of this reconstructed tree, 38 (39–1) SSD and SAD pairs of contrasts could be computed for pairs of nodes sharing an immediate common ancestor, and then re-scaled and analysed as suggested by Garland et al. [66]. Correct standardization and homogeneity of variance of standardized contrasts were confirmed using the method proposed by Purvis and Rambaut [67].

We applied a General Linear Model (GLM) treating body size as a dependent variable and species and sex as fixed factors to test for sex differences in mean body length. We tested Rensch’s rule in lineages with mixed SSD and female-biased SSD. We used the log-scaled size of one sex regressing against the log-scaled size of the other sex to test for allometry vs. isometry. When performing a simple regression between log_10_–transformed female and male size, measurement error will be approximately equal in both sexes. The Model I regression (Ordinary Least Squares, OLS) would be statistically incorrect as neither male nor female size measurements are fixed nor measured with error [2]. We therefore regarded a Model II regression (e.g. Reduced Major Axis, RMA) between log_10_ (female size) and log_10_ (male size) as more appropriate. To test whether the size relationship between sexes departed from isometry, we calculated the 95% confidence interval (CI) of the slope of the regression between their respective size contrasts and assessed whether a slope of unity fell within its bounds. All statistical tests were two-tailed, and performed on the log-transformed values of the original data.

## Competing interests

The authors have declared that no competing interests exist.

## Authors’ contributions

WBL and YZ carried out the analyses and drafted the manuscript. WBLand CQZ designed the study. RJ helped draft the manuscript. All the authors read and approved the final manuscript.

## Supplementary Material

Additional file 1: Table S1 Species, location, mean size and age within each sex and references of published papers and unpublished data for the 39 anurans species considered in this study. * indicate mean ± SE. Click here for file
