# Supplementary material for: Sexual size dimorphism in anurans fails to obey Rensch’s rule
Source: Front Zool. 2013 Mar 9;10:10. doi: 10.1186/1742-9994-10-10 (PMC3599542; doi:10.1186/1742-9994-10-10)
Supplement: Additional file 1: Table S1 — Species, location, mean size and age within each sex and references of published papers and unpublished data for the 39 anurans species considered in this study. * indicate mean ± SE. [file 1742-9994-10-10-S1.doc]

| Species | Population | Sample size | Body length ( ± SD) | | Age ( ± SD) | | References |
| --- | --- | --- | --- | --- | --- | --- | --- |
|  |  | Male/female | male | female | male | female |  |
| *Bufo alvarius** | Arizona, USA | 9/9 | 117.8 ± 2.6 | 125.0 ± 4.7 | 2.8 ± 0.3 | 2.3 ± 0.3 | Sullivan & Fernandez (1999) |
| *Bufo punctatus** | Arizona, USA | 25/8 | 52.0 ± 0.5 | 59.2± 2.0 | 2.3 ± 0.3 | 2.0 ± 0.4 | Sullivan & Fernandez (1999) |
| *Bufo cognatus** | Arizona, USA | 6/8 | 64.0 ± 5.2 | 73.6± 1.7 | 2.5 ± 0.6 | 3.4 ± 0.6 | Sullivan & Fernandez (1999) |
| *Bufo cognatus** | Arizona, USA | 15/2 | 70.4 ± 1.3 | 70.0 ± 1.5 | 3.2 ± 1.1 | 3.3± 0.7 | Sullivan & Fernandez (1999) |
| *Bufo bufo* | Netherlands | 155/53 | 52.9 ± 3.9 | 64.9 ± 4.9 | 4.3 | 5.3 | Hemelaar (1988) |
| *Bufo bufo* | Germany | 241/122 | 56.7 ± 3.9 | 68.5 ± 4.2 | 4.3 | 5.4 | Hemelaar (1988) |
| *Bufo bufo* | France | 133/95 | 68.8 ± 4.3 | 87.4 ± 8.2 | 5.2 | 6.8 | Hemelaar (1988) |
| *Bufo bufo* | Norway | 208/84 | 58.9 ± 2.9 | 72.1 ± 3.5 | 7.3 | 8.8 | Hemelaar (1988) |
| *Bufo bufo* | Switzerland | 82/34 | 62.5 ± 2.6 | 73.9 ± 3.5 | 8.6 | 9.9 | Hemelaar (1988) |
| *Bufo bufo* | Zuce, Serbia | 28/47 | 70.4 | 96.3 | 5.5 ± 1.2 | 5.9 ± 1.0 | Cvetković et al. (2009) |
| *Bufo bufo* | Trešnja, Serbia | 66/50 | 67.2 | 92.8 | 5.0 ± 1.3 | 5.4 ± 1.4 | Cvetković et al. (2009) |
| *Bufo americanus* | Illinois, USA | 34/37 | 64.3 | 71.5 | 2.6 | 3.5 | Acker et al. (1986) |
| *Bufo andrewsi* | Lingguan, China | 47/32 | 72.9 ± 3.8 | 94.2 ± 5.2 | 2.0 ± 0.8 | 3.3 ± 0.9 | Liao & Lu (2012) |
| *Bufo andrewsi* | Muping, China | 46/17 | 74.1 ± 4.3 | 98.3 ± 4.0 | 2.1 ± 0.9 | 3.6 ±1.1 | Liao & Lu (2012) |
| *Bufo andrewsi* | Yanjing, China | 18/8 | 78.2 ± 3.8 | 98.8 ± 4.9 | 2.7 ± 0.9 | 4.1 ± 0.9 | Liao & Lu (2012) |
| *Bufo andrewsi* | Dengcigou, China | 261/192 | 79.9 ± 4.6 | 99.7 ± 4.9 | 3.2 ± 1.9 | 4.2 ± 1.1 | Liao & Lu (2012) |
| *Bufo andrewsi* | Zhalangou, China | 29/9 | 80.2 ± 3.9 | 101.0 ± 3.8 | 3.3 ± 1.0 | 4.3 ± 1.4 | Liao & Lu (2012) |
| *Bufo andrewsi* | Church, China | 30/22 | 82.1 ± 3.2 | 103.2 ± 4.4 | 4.0 ± 1.0 | 5.2 ± 1.2 | Liao & Lu (2012) |
| *Bufo gargarizans* | Nanchong, China | 128/79 | 101.1 ± 5.5 | 92.9 ± 4.6 | 3.2 ± 1.2 | 3.5 ± 1.3 | Unpublished data |
| *Alytes obstetricans* | Huesca | 59/31 | 41.7 ± 4.0 | 49.7 ± 4.9 | 2.3 | 3.7 | Márquez et al. (1997) |
| *Alytes cisternasii* | Merida | 74/13 | 35.8 ± 2.3 | 38.6 ± 2.7 | 2.4 | 3.2 | Márquez et al. (1997) |
| *Pelobates fuscus* | France | 87/29 | 45.9 | 53.9 | 3.6 ± 0.8 | 4.6 ± 1.7 | Eggert & Guyétant (1999) |
| *Xenopus laevis** | Wales, UK | 51/64 | 59.9 ± 0.5 | 63.3 ± 0.7 | 3.7 ± 0.1 | 3.9 ± 0.2 | Measey (2001) |
| *Mantidactylus microtympanum* | Madagascar | 26/33 | 74.7 ± 8.2 | 94.1 ± 3.2 | 4.1 ± 1.1 | 4.9 ± 1.9 | Guarino et al*.* (1998) |
| *Scaphiopus couchii** | Arizona, USA | 23/6 | 52.4 ± 0.7 | 50.4 ± 1.2 | 2.1 ± 0.2 | 2.3 ± 0.3 | Sullivan & Fernandez (1999) |
| *Hyla arborea* | Germany | 94/49 | 43.1 ± 2.5 | 44.4 ± 2.8 | 3.1 ± 1.2 | 3.3 ± 1.1 | Friedl & Klump (1997) |
| *Hyla crucifer* | Maryland, USA | 184/59 | 26.5 ± 2.1 | 28.4 ±3.2 | 2.6 ± 1.1 | 2.8 ± 0.9 | Lykens & Forester (1987) |
| *Hyla annectans* | Baoxing, China | 31/17 | 34.0 ± 1.5 | 40.9 ± 1.4 | 2.9 ± 0.7 | 3.3 ± 0.7 | Liao & Lu (2010a) |
| *Hyla annectans* | Baoxing, China | 33/16 | 31.9 ± 1.4 | 38.1 ± 1.0 | 2.1 ± 0.6 | 2.9 ± 0.8 | Liao & Lu (2010a) |
| *Pelophylax nigromaculata** | Nanchong, China | 79/60 | 66.0 ± 0.9 | 71.2 ± 1.7 | 2.8 ± 0.1 | 3.0 ± 0.2 | Mao et al. (2012) |
| *Pelophylax nigromaculata** | Baoxing, China | 32/32 | 66.6 ± 0.7 | 75.7 ± 0.4 | 2.4 ± 0.1 | 2.8 ± 0.1 | Liao et al. (2010) |
| *Pelophylax nigromaculata** | Suining, China | 24/22 | 72.2 ± 0.6 | 83.9 ± 1.2 | 2.4 ± 0.1 | 3.0 ± 0.1 | Liao et al. (2010) |
| *Pelophylax nigromaculata** | Shizuhara, Japan | 72/24 | 66.7 ± 1.2 | 76.3 ± 3.0 | 3.4 ± 0.2 | 4.1 ± 0.3 | Khonsue et al. (2001) |
| *Pelophylax nigromaculata* | Katata, Japan | 62/10 | 63.6 ± 2.0 | 75.9 ± 4.0 | 3.3 ± 0.3 | 4.1 ± 0.6 | Khonsue et al. (2001) |
| *Pelophylax ridibunda* | Thrace, Greece | 52/56 | 69.0 ± 12.6 | 82.4 ± 13.3 | 3.0 ± 1.0 | 3.7 ± 1.1 | Kyriakopoulou-Sklavounou et al. (2008) |
| *Pelophylax pleuraden* | Lingnan, China | 36/27 | 45.4 ± 4.7 | 50.3 ± 8.1 | 2.9 ± 0.7 | 3.0 ± 0.8 | Lou et al. (2012) |
| *Pelophylax pleuraden* | Lingnan, China | 24/18 | 44.4 ± 1.6 | 48.5 ± 3.2 | 2.7 ± 0.4 | 2.5 ± 0.6 | Lou et al. (2012) |
| *Pelophylax pleuraden* | Lingnan, China | 21/15 | 45.0 ± 1.9 | 50.4 ± 6.3 | 2.8 ± 0.7 | 3.1 ± 0.7 | Lou et al. (2012) |
| *Pelophylax pleuraden* | Lingnan, China | 44/8 | 41.4 ± 3.8 | 46.7 ± 5.5 | 1.7 ± 0.4 | 2.0 ± 0.8 | Lou et al. (2012) |
| *Pelophylax pleuraden* | Lingnan, China | 22/17 | 44.8 ± 2.3 | 51.8 ± 3.1 | 2.7 ± 0.4 | 3.4 ± 0.9 | Lou et al. (2012) |
| *Rana muscosa* | Sierra Nevada | 44/74 | 56.1 ± 1.3 | 63.6 ± 1.0 | 4.0 ± 0.3 | 4.1 ± 0.3 | Matthews & Miaud (2007) |
| *Rana omeimontis* | Yibin, China | 63/64 | 49.2 ± 4.1 | 51.4 ± 5.6 | 1.2 ± 0.9 | 2.1 ± 0.8 | Liu et al. (2012) |
| *Rana ridibunda* | Turkey | 38/11 | 64.6 ± 11.1 | 74.6 ± 13.4 | 3.9 ± 1.4 | 3.7 ± 1.0 | Yilmaz et al. (2005) |
| *Rana epeirotica* | Ioannina, Greece | 145/173 | 72.7 ±8.0 | 83.6 ±11.7 | 2.8 ± 0.9 | 3.2 ± 0.8 | Tsiora & Kyriakopoulou-Sklavounou (2002) |
| *Rana temporaria* | Italy | 78/21 | 79.5 ± 7.8 | 86.1 ± 13.8 | 4.8 ±2.1 | 6.0 ± 2.1 | Guarino et al. (2008) |
| *Rana temporaria* | Swiss Alps | 119/88 | 73.7 ± 3.1 | 81.6 ± 5.4 | 4.4 ± 1.3 | 4.7 ± 1.5 | Ryser (1988) |
| *Rana temporaria* | French Alps | 30/32 | 77.8 ± 3.0 | 83.6± 2.1 | 8.3 ± 3.0 | 8.4 ± 2.2 | Miaud *et al.* (1999) |
| *Rana sylvatica* | Queébec, Canada | 56/23 | 43.6 ± 2.6 | 49.3 ± 2.5 | 2.8 ± 0.7 | 3.2 ± 0.7 | Bastien & Leclair (1992) |
| *Rana sylvatica* | Virginia, USA | 462/260 | 55.3 ± 3.1 | 64.4 ± 3.5 | 2.9 ± 0.4 | 3.6 ± 0.5 | Berven (1982) |
| *Rana sylvatica** | Maryland, USA | 194/86 | 41.7 ± 3.7 | 47.7 ± 3.7 | 1.2 ±0.4 | 2.0 ± 0.1 | Berven (1982) |
| *Rana sylvatica* | Queébec, Canada | 179/33 | 43.6 ± 2.0 | 48.8 ± 2.7 | 2.5 ± 0.7 | 2.8 ± 0.8 | Sagor *et al.* (1998) |
| *Rana chensinensis* | Shanxi, China | 27/21 | 39.5 | 45.0 | 1.9 | 2.7 | Lu et al.(2006) |
| *Rana chensinensis* | Shanxi, China | 22/20 | 42.5 | 44.0 | 2.1 | 2.6 | Lu et al.(2006) |
| *Rana chensinensis* | Shanxi, China | 24/20 | 43.5 | 50.5 | 2.4 | 3.9 | Lu et al.(2006) |
| *Rana chensinensis** | Shanxi, China | 236/86 | 41.8 ± 4.1 | 43.7 ± 5.8 | 1.4 ± 0.1 | 1.7 ± 0.2 | Ma et al. (2009a) |
| *Rana chensinensis** | Shanxi, China | 38/14 | 42.9 ± 3.3 | 50.5 ± 5.7 | 1.3 ± 0.1 | 3.4 ± 0.3 | Ma et al. (2009a) |
| *Rana chensinensis** | Shanxi, China | 103/49 | 47.0 ± 3.2 | 52.5 ± 5.6 | 2.0 ± 0.2 | 2.8 ± 0.3 | Ma et al. (2009a) |
| *Rana chensinensis* | Liaoning, China | 63/60 | 60.6 ± 2.9 | 67.7 ± 4.6 | 2.2 ± 0.9 | 2.4 ± 0.9 | Chen et al. (2011) |
| *Rana amurensis* | Inner Mongolia, China | 45/46 | 45.9 ± 5.8 | 41.5 ± 5.3 | 1.8 ± 1.0 | 1.6 ± 0.8 | Liao (2011) |
| *Rana cascadae* | Oregon, USA | 539/227 | 44.3± 2.6 | 39.8 ± 3.4 | 2.0 ± 0.9 | 1.5 ± 0.7 | Briggs & Storm (1970) |
| *Rana catesbeiana* | Canada |  | 105.3 | 113.2 | 4.4 | 5.3 | Shirose *et al.* (1993) |
| *Fejervarya limnocharis* | Lingguan, China | 150/45 | 41.2 ± 2.3 | 45.5 ± 2.3 | 1.6 ± 0.7 | 2.2 ± 0.9 | Liao et al. (2011) |
| *Fejervarya limnocharis* | Suining, China | 22/20 | 38.8 ± 1.8 | 47.3 ± 2.8 | 1.6 ± 0.6 | 2.1 ± 0.7 | Liao et al. (2011) |
| *Amolops mantzorum* | Baoxing, China | 52/21 | 54.2 ± 2.3 | 69.5 ± 2.8 | 4.0 ±1.2 | 4.7 ± 1.5 | Liao & Lu (2010b) |
| *Amolops mantzorum* | Baoxing, China | 42/24 | 52.4 ± 2.3 | 67.1 ± 3.0 | 3.9 ± 1.1 | 4.2 ± 1.1 | Liao & Lu (2010b) |
| *Amolops mantzorum* | Baoxing, China | 76/67 | 54.7 ± 2.6 | 69.0 ± 2.9 | 4.3 ± 1.3 | 5.2 ± 1.4 | Liao & Lu (2010c) |
| *Amolops lifanensis* | Sichuan, China | 20/30 | 50.7 ± 4.2 | 68.9 ± 4.3 | 3.8 ± 1.2 | 4.7 ± 1.6 | Liu et al. (2011) |
| *Rhacophorus omeimontis* | Baoxing, China | 141/28 | 64.7 ± 2.4 | 76.7 ± 3.1 | 3.6 ± 1.0 | 4.3 ± 1.2 | Liao & Lu (2011) |
| *Rhacophorus omeimontis* | Baoxing, China | 23/8 | 62.2 ± 1.8 | 75.6 ± 1.8 | 2.8 ± 0.8 | 3.4 ± 0.9 | Liao & Lu (2011) |
| *Rhacophorus omeimontis* | Baoxing, China | 39/15 | 60.9 ± 1.3 | 74.9 ± 2.9 | 2.3 ± 0.8 | 3.1 ± 0.8 | Liao & Lu (2011) |
| *Rhacophorus megacephalus* | Guizhou, China | 28/3 | 45.7 ± 3.4 | 61.9 ± 6.1 | 2.2 ± 0.9 | 2.3 ± 1.5 | Unpublished data |
| *Rhacophorus megacephalus* | Guangxi, China | 36/6 | 50.6 ± 2.8 | 65.6 ± 2.8 | 3.2 ± 0.8 | 3.2 ± 0.9 | Unpublished data |
| *Rhacophorus megacephalus* | Guangxi, China | 44/12 | 58.7 ± 3.6 | 80.7 ± 4.0 | 3.1 ± 0.9 | 3.3 ± 1.0 | Unpublished data |
| *Hylarana guentheri* | Suining, China | 86/79 | 67.8 ± 3.7 | 75.3 ± 4.1 | 2.4 ± 0.7 | 3.2 ± 1.1 | Li et al. (2010) |
| *Nanorana parkeri* | central Tibet, China | 138/73 | 39.4 | 45.4 | 4.5 | 4.9 | Ma et al. (2009b) |
| *Nanorana parkeri* | central Tibet, China | 335/204 | 36.1 | 42.6 | 4.7 | 5.4 | Ma et al. (2009b) |
| *Nanorana parkeri* | central Tibet, China | 754/599 | 36.5 | 41.3 | 5.8 | 6.3 | Ma et al. (2009b) |
| *Nanorana parkeri* | central Tibet, China | 128/111 | 37.4 | 40.5 | 6.0 | 7.0 | Ma et al. (2009b) |
| *Mixophyes fasciolatus** | Queensland, Australian | 34/25 | 67.3 ± 2.2 | 72.5 ± 5.4 | 3.5 ± 0.1 | 3.8 ± 0.2 | Morrison (2001) |
| *Mixophyes fasciolatus** | Queensland, Australian | 17/1 | 63.2 ± 1.8 | 78.1 | 3.5± 0.1 | 4.3± 0.3 | Morrison (2001) |
| *Mixophyes fasciolatus** | Queensland, Australian | 56/30 | 64.3 ± 3.7 | 74.9 ± 6.2 | 3.7± 0.1 | 4.0 ± 0.1 | Morrison (2001) |
| *Mixophyes fleayi** | Queensland, Australian | 40/9 | 62.6 ± 4.6 | 69.7 ± 4.7 | 3.9 ± 0.1 | 4.3 ± 0.3 | Morrison (2001) |
| *Odorrana grahami* | Lingnan, China | 28/28 | 71.5 ± 6.0 | 86.7 ± 13.2 | 3.5 ± 0.9 | 3.8 ± 1.1 | Unpublished data |
| *Odorrana grahami* | Lingnan, China | 28/15 | 67.4 ± 9.5 | 71.7 ± 15.4 | 3.3 ± 1.1 | 3.6 ± 1.4 | Unpublished data |
| *Odorrana grahami* | Lingnan, China | 17/23 | 69.3 ± 6.7 | 58.1 ± 15.4 | 2.3 ± 1.3 | 3.6 ± 1.1 | Unpublished data |
| *Odorrana grahami* | Lingnan, China | 35/27 | 67.2 ± 5.8 | 76.1 ± 8.7 | 3.1 ± 1.1 | 2.8 ± 1.0 | Unpublished data |

Additional table 1 Species, location, mean size and age within each sex and references of published papers and unpublished data for the 39 anurans species considered in this study. * indicate mean ± SE.

References

Acker PM, Kruse KC, Krehbiel EB: **Aging *Bufo americanus* by skeletochronology**. *J Herpetol* 1986, **20**: 570–574.

Bastien H, Leclair Jr R: **Aging wood frogs (*Rana sylvatica*) by skeletochronology.** *J Herpetol* 1992, **26**: 222–225.

Berven KA: **The genetic basis of altitudinal variation in the wood frog, *Rana sylvatica*. I. An experimental analysis of life-history traits**. *Evolution* 1982, **36**: 962–983.

Briggs JL, Storm RM: **Growth and population structure of the cascade frog, *Rana cascadae* Slater**. *Herpetologica* 1970, **26**: 283–300.

Chen BY, Liao WB, Mi ZP: **Body size and age of the China Wood Frog (*Rana chensinensis*) in northeastern China**. *NW J Zool* 2011, **7**: 236–242.

Cvetković D, Tomašević N, Ficetola G F, Crnobrnja-Isailović J, Miaud C: **Bergmann’s rule in amphibians: combining demographic and ecological parameters to explain body size variation among populations in the common toad *Bufo bufo*.** [*J Zool Syst Evol Res*](http://www.wiley.com/bw/submit.asp?ref=0947-5745) 2009, **47**: 171–180.

Eggert C, Guyétant R: **Age structure of a spadefoot toad *Pelobates fuscus* (Pelobatidae) population**. Copeia 1999, **1999**: 1127–1130.

Friedl TWP, Klump GM: **Some aspects of population biology in the European treefrog, *Hyla arborea*.** *Herpetologica* 1997, **53**: 321–330.

Guarino FM, Andreone F, Angelini F: **Growth and longevity by skeletochronological analysis in *Mantidactylus* *microtympanum*, a rain-forest anuran from southern Madagascar.** Copeia 1998, 1**998**: 194–198.

Guarino FM, Erismis UC: **Age determination and growth by skeletochronology of *Rana holtzi*, an endemic frog from Turkey**. *Ital J Zoo*l 2008, **73**: 237–242.

Hemelaar ASM: **Age, growth and other population characteristics of *Bufo bufo* from different latitudes and altitudes**. *J Herpetol* 1988, **22**: 369–388.

Khonsue W, Matsui M, Hirai T, Misawa Y: **A comparison of age structures in two populations of a pond frog *Rana nigromaculata* (Amphibia: Anura)**. *Zool Sci* 2001, **18**: 597–603.

Kyriakopoulou-Sklavounou P, Stylianou P, Tsiora A: **A skeletochronological study of age, growth and longevity in a population of the frog *Rana ridibunda* from southern Europe**. *Zoology* 2008, **111**: 30–36.

Li C, Liao WB, Yang ZS, Zhou CQ: **A skeletochronological estimation of age structure in a population of the Guenther’s frog, *Hylarana guentheri*, from western China**. *Acta Herpetol* 2010, 5: 1-11.

Liao W B, Zhou C Q, Yang Z S, Lu X : Age, size and growth in two populations of the dark-spotted frog *Rana nigromaculata* at different altitudes in southwestern China. *Herpetol J* 2010, **20**: 77–82.

Liao WB: **A skeletochronlogical estimate of age in a population of the** **Siberian Wood Frog, *Rana amurensis*, from northeastern Chin**a. *Acta Herpetol* 2011, **6**: 237–245.

Liao WB, Lu X: Age structure and body size of the Chuanxi tree toad *Hyla annectans chuanxiensis* from two different elevations (China). *Zool Anz* 2010a, 248: 255–263.

Liao WB, Lu X: **A skeletochronological estimation of age and body size by the Sichuan torrent frog (*Amolops mantzorum*) between two populations at different altitudes**. *Anim Biol* 2010b**, 60**: 479–489.

Liao WB, Lu X: **Age and growth of a subtropical high-elevation torrent frog, *Amolops mantzorum*, in western China**. *J Herpetol* 2010c, 44: 172–176.

Liao WB, Lu X: **Variation in body size, age and growth in a subtropical treefrog (*Rhacophorus omeimontis*) along an altitudinal gradient in western China**. *Ethol Ecol Evol* 2011, **23**: 248–261.

Liao WB, Lu X: **Adult body size = *f* (initial size + growth rate × age): explaining the proximate cause of Bergman’s cline in a toad along altitudinal gradients**. *Evolu Ecol* 2012, **26**: 579–590.

Liao WB, Lu X, Shen YW, Hu JC: **Age structure and body size of two populations of the rice frog *Rana limnocharis* from different altitudes**. *Ital J Zool* 2011, 78: 215–221.

Liu YH, Liao WB, Zhou CQ, Mi ZP, Mao M: **Age structure of *Amolops lifanensis****. J China West Norm Univ (Nat Sci)* 2011, **23**: 151–155.

# Lou SL, Jin L, Liu YH, Mi ZP, Tao G, Tang YM, Liao WB: Altitudinal variation in age and body size in Yunnan Pond Frog (*Pelophylax pleuraden*). *Zool Sci* 2012, 29: 493–498.

Lu X, Li B, Liang JJ: **Comparative demography of a temperate anuran, *Rana chensinensis*, along a relatively fine altitudinal gradient.** [*Can J Zool*](http://proquest.umi.com/pqdweb?RQT=318&pmid=36147&TS=1199187314&clientId=26439&VInst=PROD&VName=PQD&VType=PQD) 2006, **84**: 1789–1795.

Lykens DV, Forester DC: **Age structure in the spring peeper: do males advertise longevity?** *Herpetologica* 1987, **43**: 216–223.

Ma XY, Tong LN, Lu X: **Variation of body size, age structure and growth of a temperate frog, *Rana chensinensis*, over an elevational gradient in northern China***. Amphibia-Reptilia* 2009, **30**: 111–117.

Marquez R, Esteban M, Castanet J: **Sexual size dimorphism in midwife toads *Alytes obstetricans* and *A. cisternasii*.** *J Herpetol* 1997, **31**: 52–59.

Matthews KR, Miaud C: **A skeletochronological study of the age structure, growth, and longevity of the mountain yellow-legged frog, *Rana muscosa*, in the Sierra Nevada, California**. *Copeia* 2007, **2007**: 986–993.

Measey GJ: **Growth and ageing of feral *Xenopus laevis* (Daudin) in South Wales, UK**. *J Zool* 2001, **254**: 547–555.

Miaud C, Guyétant R, Elmberg J: **Variations in life history traits in the common frog *Rana temporaria* (Amphibia: Anura): a literature review and new data from the French Alps**. *J Zool* 1999, **249**: 61–73.

Morrison FC: **Altitudinal Variation in the Life History of Anurans in Southeast Queensland**. Unpublished Ph.D., Griffith University, Bundall, Queensland, Australia. 2001.

Ryser J: **Determination of growth and maturation in the common frog, *Rana temporaria*, by skeletochronology**. *J Zool* 1988, **216**: 673–685.

Sagor ES, Ouellet M, Barten E, Green DM: **Skeletochronology and geographic variation in age structure in the wood frog, *Rana sylvatica*.** *J Herpetol* 1998, **32**: 469–474.

Shirose LJ, Brooks RJ, Barta JR, Desser SS: **Intersexual differences in growth, mortality, and size at maturity in bullfrogs in central Ontario**. *Can J Zool* 1993, **71**: 2363–2369.

Sullivan BK, Fernandez PJ: **Breeding activity, estimated age-structure, and growth in sonoran desert anurans.** *Herpetologica* 1999, **55**: 334–343.

Tsiora A, Kyriakopoulou-Sklavounou P: **A skeletochronological study of age and growth in relation to adult size in the water frog *Rana epeirotica***. *Zoology* 2002, **105**: 55–60.

Yilmaz N, Kutrup B, Cobanoglu U, Ozoran Y: **Age determination and some parameters of a *Rana ridibunda* population in Turkey***. Acta Zool Acad Sci Hung* 2005, **51**: 67–74.
